# The Flavonoid Pathway Regulates the Petal Colors of Cotton Flower

**DOI:** 10.1371/journal.pone.0072364

**Published:** 2013-08-12

**Authors:** Jiafu Tan, Maojun Wang, Lili Tu, Yichun Nie, Yongjun Lin, Xianlong Zhang

**Affiliations:** National Key Laboratory of Crop Genetic Improvement, Huazhong Agricultural University, Wuhan, Hubei, China; New Mexico State University, United States of America

## Abstract

Although biochemists and geneticists have studied the cotton flower for more than one century, little is known about the molecular mechanisms underlying the dramatic color change that occurs during its short developmental life following blooming. Through the analysis of world cotton germplasms, we found that all of the flowers underwent color changes post-anthesis, but there is a diverse array of petal colors among cotton species, with cream, yellow and red colors dominating the color scheme. Genetic and biochemical analyses indicated that both the original cream and red colors and the color changes post-anthesis were related to flavonoid content. The anthocyanin content and the expression of biosynthesis genes were both increased from blooming to one day post-anthesis (DPA) when the flower was withering and undergoing abscission. Our results indicated that the color changes and flavonoid biosynthesis of cotton flowers were precisely controlled and genetically regulated. In addition, flavonol synthase (*FLS)* genes involved in flavonol biosynthesis showed specific expression at 11 am when the flowers were fully opened. The anthocyanidin reductase (*ANR)* genes, which are responsible for proanthocyanidins biosynthesis, showed the highest expression at 6 pm on 0 DPA, when the flowers were withered. Light showed primary, moderate and little effects on flavonol, anthocyanin and proanthocyanidin biosynthesis, respectively. Flavonol biosynthesis was in response to light exposure, while anthocyanin biosynthesis was involved in flower color changes. Further expression analysis of flavonoid genes in flowers of wild type and a flavanone 3-hydroxylase *(F3H)* silenced line showed that the development of cotton flower color was controlled by a complex interaction between genes and light. These results present novel information regarding flavonoids metabolism and flower development.

## Introduction

The diversity of flower color is one of the most beautiful gifts of nature, and it serves as a versatile tool for plant biochemists, geneticists and ecologists. Different flower colors are constituted with various pigments and co-pigments. The betalains, carotenoids, and anthocyanins are the three major, well-characterized groups of pigments [1]. Flavonols, pH and ions also play important roles in determining flower color [2], [3], [4]. In addition to endogenous genetic effects, environmental factors such as light and temperature could also be responsible for the color of flowers [5], [6], [7], [8]. In addition to its general phenotype, color also confers flowers with diverse biological functions, such as protection against UV-light and the attraction of pollinators [9], [10]. Within a single flower, color changes during flower development are widespread throughout the angiosperms [11], [12]. Multiple studies have indicated that, although it is important for pollinator interaction, the mechanism of flower color changes pre- and post- anthesis is not well understood [13], [14], [15].

Flavonoids, particularly anthocyanidin glycosides, are the major flower pigments [16]. The metabolism of the flavonoid pathway has been well analyzed in *Petunia*, *Arabidopsis* and *Antirrhinum* [17], [18], [19]. In most cases, anthocyanin accumulation was tightly linked with flower development and color changes [20]. The anthocyanin biosynthesis pathway has been mostly studied, and the biosynthesis of flavonol, which together with the biosynthesis of anthocyanin comprising the major biosynthesis pathways in flavonoid metabolism, has also been well studied in flower development due to its high antioxidation and UV protection [10]. Flavonol and anthocyanin metabolism can be induced by light [21], [22]. Flavonoids are derived from phenylalanine, catalyzed by phenylalanine ammonia-lyase (PAL). Then it was mediated by a common step with chalcone synthase (CHS), flavanone 3-hydroxylase (F3H), and fluxed into anthocyanin biosynthesis by dihydroflavonol 4-reductase (DFR), anthocyanidin synthase (ANS) and UDP-glucose: flavonoid 3-Oglucosyltransferase (UFGT); fluxed into flavonol biosynthesis by flavonol synthase (FLS) following F3H; or fluxed into proanthocyandin biosynthesis through anthocyanidin reductase (ANR) following ANS [23]. Most flavonoid-related genes showed co-regulation and co-expression in plants [24], [25], [26]. The expression of these genes was mostly light dependent [22], [27], [28], while a light independent mechanism impacting flavonoids biosynthesis was also observed [29]. Various interactions were observed between different enzymes in *Arabidopsis*, which indicates that these enzymes function as a complex [30], [31], [32].

Cotton (*Gossypium spp.*) is composed of approximately 50 species, including approximately 5 allotetraploid species and 45 diploids [33]. Many phenotypic polymorphisms exist among these species, of which the variety of flower color is one of the more obvious, and color variety had also been used in modern genetic study [34]. The biochemical study of cotton flower color has more than a century of history [35]. The color of the cotton flower is due to the effects of both flavonol and anthocyan [36]. Quercetin and cyanidin have been shown to be the major flavonol and anthocyan compounds, respectively, throughout the genus [35], [37]. Varity, environment and fertilization could affect the composition of cotton flower pigments [35], [38]. However, the pigment of the cotton flower is less affected by environmental factors than that of the leaf [39], and it could be stable inheritance [40]. The flavonoids in the cotton flower are kinds of biologically-active chemicals and show insect resistance and antibacterial properties [41], [42]. The color of the cotton flower has also been employed as a tool for the genetic and taxonomic study of cotton [43], [44], [45]. Thousands of records of cotton flower color have been observed and presented in the international germplasm systems; however, these records all represent only one time-point phenotype. Cotton flowers suffer from a short life, as they bloom in the morning and begin to wither in the afternoon of the day of anthesis and abscise near noon of the next day under common field growth conditions; the colors dramatically change during flower development [35], [38].

Here, we present data obtained through biological analysis showing that the color of the cotton flower is primarily composed of flavonoids. The change in color is associated with the expression of flavonoid genes, particularly those involved in anthocyanin biosynthesis. Light plays an important role in pigment accumulation and flavonoid biosynthesis, but an inherent factor also exists in the flavonoid pathway during flower development.

## Materials and Methods

### Plant materials

The materials were collected from the experimental field of Huazhong Agricultural University, Wuhan, China. No specific permissions were required for these locations or activities. It did not involve endangered or protected species. The cotton plants used in this study, *Gossypium hirsutum* YZ1, an F3H stable silence line of YZ1 (f3h) which was generated by our laboratory [46], brown cotton *G. hirsutum* T586, and *G. barbadense* 3-79, were cultivated with standard farming practices and management. Flowers were harvested on a typical sunny day in August at different time points ranging from the day before blooming to the day after blooming. The day of anthesis was noted as 0 DPA. Different time points were noted according to Beijing time (GMT +8). Flowers at six time points including 6, 11 and 12 am, 3 and 6 pm on 0 DPA and 6 am on 1 DPA, were collected for analysis. The anther and pistils were carefully removed to avoid contamination. Flowers were then cut flush with the margin of the sepal, weighed and either immediately ground into powder in liquid nitrogen or immersed in liquid nitrogen and stored at –70°C until use.

### Cotton germplasm analysis

To collect information about the cotton flower, different Internet germplasm resources were consulted. Two of them, the Chinese Crop Germplasm Resources Information System (CGRIS) (http://icgr.caas.net.cn/#) and the National Plant Germplasm System (GRIN) (http://www.ars-grin.gov/npgs/searchgrin.html) containing abundant cotton petal data were referred to for analysis. Several representative pictures of cotton flower were obtained from the Internet (including http://www.learnnc.org/lp/multimedia/9447, http://commons.wikimedia.org/wiki/File:Darwins-cotton-flower.jpg, http://www.photostuff. org/galapagos3.html, http://www.alohafriendsphotos.com/flowers2.html).

### Quantification of anthocyanins

Petals were weighed and ground in liquid nitrogen, each flower was considered to be an individual sample. Anthocyanins were extracted from the flowers in acidic (1% HCl [w/v]) methanol for 48 h. The extraction was performed with a ratio of 10 ml buffer to 1 g sample. Anthocyanin was quantified by measuring the absorbance at 530 nm [47]. For each sample, more than five flowers were used for analysis.

### Photography and image management

The flowers were collected from the experimental field and immediately photographed with a digital camera. Flowers that were harvested at the same time point were taken into one photo, and identical background and camera parameters were used at each time point. Photos of flowers taken at different time points were combined into one image with Adobe Photoshop 7.0.

### Flower shading and emasculation treatment

The flowers were covered with a kraft bag before anthesis in the afternoon of -1 DPA to avoid light exposure; and the bags were large enough to permit flower opening. Flowers from the same plant and same position were taken as control and were studied from 11 am on 0 DPA to 6 am on 1 DPA. For flower emasculation treatment, the anther and pistil of each flower were carefully removed with scissors at approximately 8 am on 0 DPA, when the petals were not fully open. Flowers from the same plants and at the same position were taken as control and analyzed at 8 am on 1 DPA. For each treatment, more than five flowers were analyzed.

### PH measurement

The pH of cotton flower was measured according to a previous report [4]. Three fresh flowers for each treatment were harvested from the field. The petals were collected, washed twice with deionized water within 15 min after removal from the plant, and then ground with a pestle and mortar for 1 min in 5 ml deionized water per flower. The pH of the homogenate was measured immediately with a pH electrode (Mettler-Toledo FE20, Shanghai).

### Quantitative real-time PCR

Cotton flowers were harvested at different time points for flavonoid gene expression analysis. RNA extraction was performed following the procedure of the Spectrum Plant Total RNA Kit of Sigma-Aldrich (USA), cDNA synthesis and qRT-PCR were performed as previously described [48], with cotton ubiquitin gene *UBQ7* as the reference gene [49]. Flavonoid gene sequences were obtained from the public NCBI UniGene data bank and the cotton D genome sequence (http://www.phytozome.com/). The expression of all genes was normalized to *UBQ7*. Three biological replicates were performed. The error bars represent the standard deviations (SD). All primers are listed in Table S1. SuperScript® III reverse transcription kits were from Invitrogen (Carlsbad, USA). Reagents (iTaq SYBR Green supermix with ROX) for real-time PCR were from Bio-Rad (Foster City, USA).

## Results

### The cotton (*Gossyoium spp.*) flower contains a variety of colors

**Figure 1:**
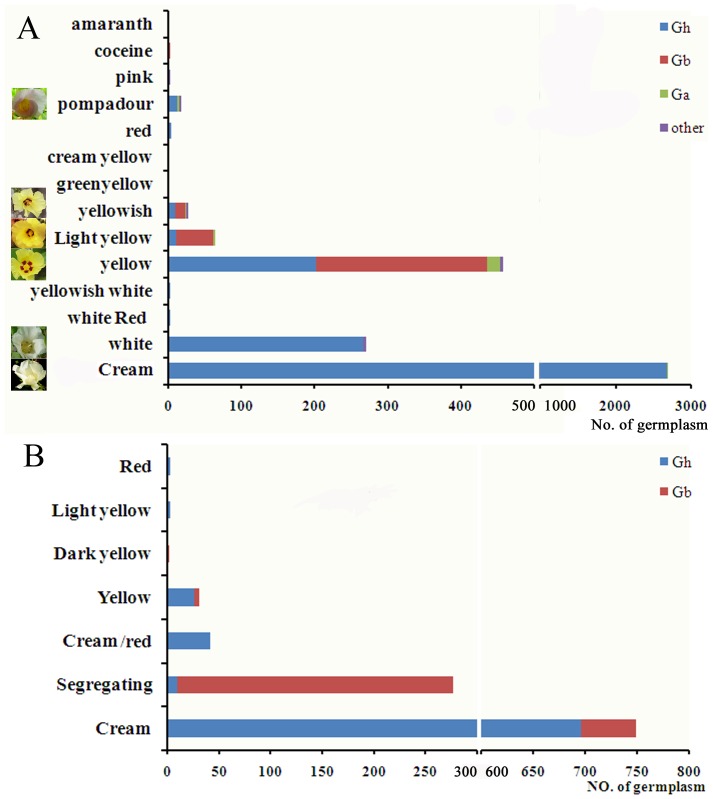
Variety of cotton flower colors obtained from the global germplasms collection systems. Germplasms from the Chinese Crop Germplasm Resources Information System (A) and the National Plant Germplasm System (B), with recorded petal colors, were analyzed. Colors were classified according to the record. *Gossypium hirsutum* (Gh), *G. barbadense* (Gb) and *G. arboreum* (Ga) were the three major cotton species in these systems. The remainders were classified as ‘other’ and mostly contained wild cotton.

The flower color is one of the most variable traits of cotton. Based on an international survey of cotton germplasms, a total of 4664 germplasms containing flower color information were identified. Among them, 3557 germplasms are from the Chinese Crop Germplasm Resources Information System (CGRIS) (Figure 1A), and the remaining 1107 germplasms are from the National Plant Germplasm System (GRIN) (Figure 1B). In total, 14 types of colors are defined in CGRIS and 7 types are done in GRIN. These colors can be divided into three major colour schemes: cream is the most common color with composing more than 70% of the germplasms in both systems; yellow is the secondary color, composing nearly 16% of the germplasms from CGRIS and 3% from GRIN it; red is the third color, although it accounts for less than 5% in both systems. There are very few white flower germplasms listed in CGRIS. Different germplasm systems show different color patterns: most of the germplasms in both systems are *Gossypium hirsutum* with approximately 76% in CGRIS and 89% in GRIN having cream flowers. White and yellow flowers are also common in *G. hirsutum*. *G. barbadense* is the second-most abundant germplasm in both systems with the primary flower color of yellow (if well defined, because there was an ambiguous definition referred to ‘segregating’ within the GRIN system that included up to 81.6% of the *G. barbadense* in that system, although these flowers appeared to be yellow in most cases, we did not include them). There are several diploid A genome and wild cotton germplasms in CGRIS having yellow flowers. It also shows that most of the red flower germplasms are *G. hirsutum*, but only one germplasm flower of *G. barbadense* is red and defined as coceine in the CGRIS.

### Cotton flower color changes dramatically after blooming

**Figure 2:**
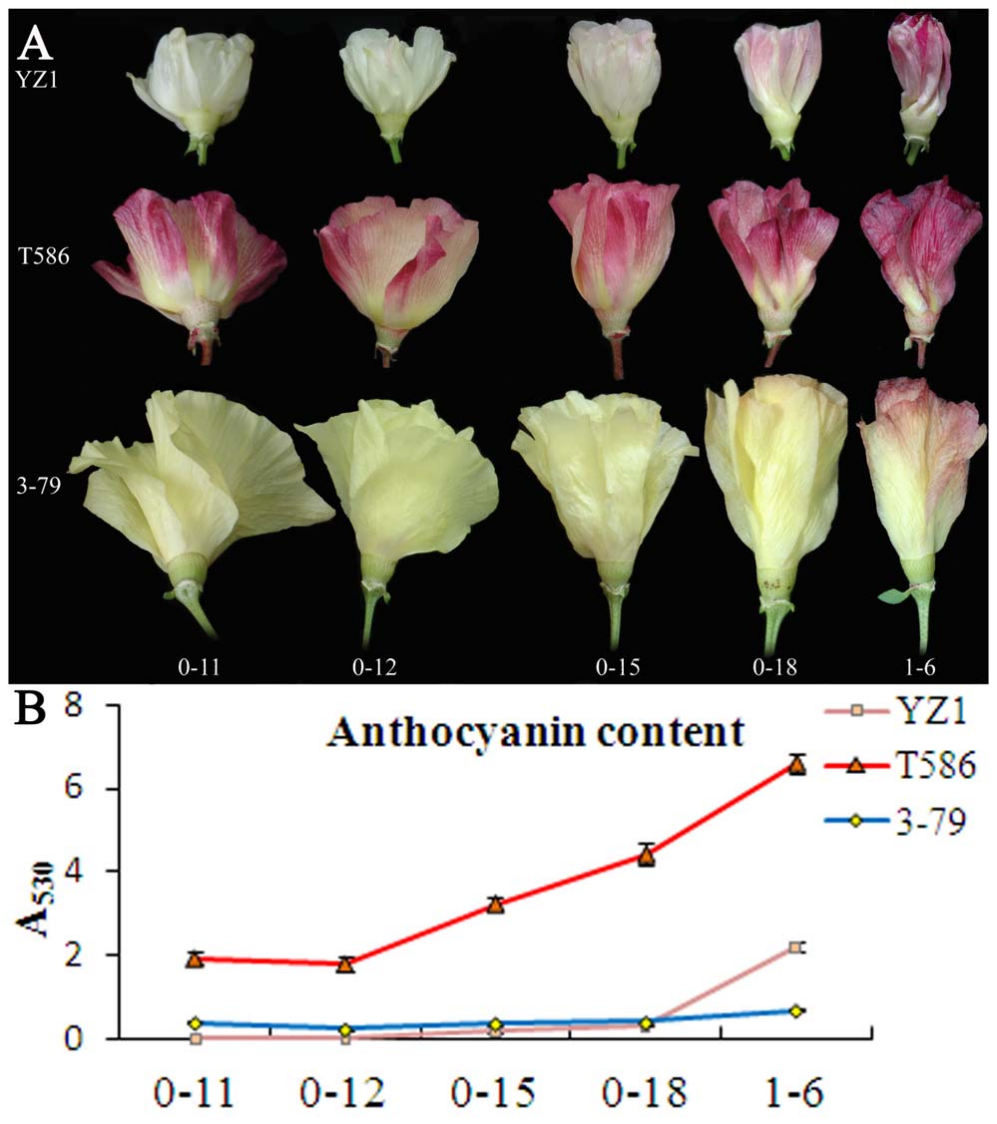
Petal colors change during flower development. A, flowers from *G. hirsutum* YZ1, T586 and *G. barbadense* 3-79 were collected from the field at 11 am, 12 am, 3 pm, and 6 pm on 0 DPA (referred as 0-11, 0-12, 0-15, and 0-18, respectively) and 6 am on 1 DPA (referred as 1-6). B, the anthocyanin contents of these flowers were measured at A_530_. Three repeats with more than five flowers for each repeat were analyzed. Error bars represent SD.

Three different flower color species were studied (Figure 2). YZ1 represents the most common cream-colored flower of *G. hirsutum*. T586 represents the red flower species, and 3-79 represents the yellow-colored flowers of *G. barbadense*. Flowers of these cotton species were collected from 11 am on the day of anthesis (0 DPA) to 6 am on the second day of anthesis (1 DPA) for analysis. All of the flowers changed the color during this period. Red pigment accumulated throughout the flower development (Figure 2A). The flower of YZ1 was cream at 11 am, and appeared a little bit red at 12 am, gradually accumulating to 6pm and then dramatically increasing color to 6 am on 1 DPA (Figure 2A). The flower of T586 was light red at 11 am, afterwards the red color deepened and the petal was dark red at 6 am on 1 DPA. Although the flower color of 3-79 was primarily yellow, red color was still emerged from the edge of the petal at 6 pm on 0 DPA and then increased further (Figure 2A). All of the flower color changes corresponded to an accumulation of anthocyanins (Figure 2B). T586 had about two times higher anthocyanin content than YZ1 and 3-79 during flower development in correspondence with its much intense color than the other species (Figure 2B). These results showed that anthocyanin biosynthesis was enhanced during flower development, conferring the petals with an intense red color. Dramatic anthocyanin accumulation occurred during the night of 0 DPA for YZ1 (Figure 2B).

### Shade reduces accumulation of anthocyanins in cotton flowers

**Figure 3:**
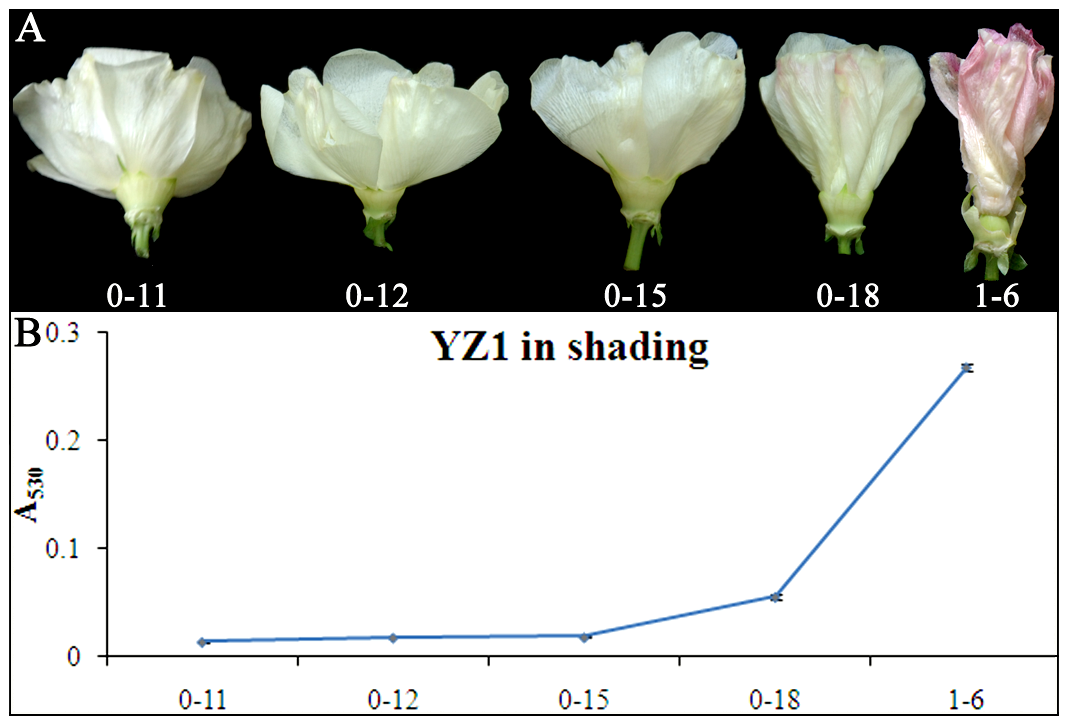
Flower color is affected by shade treatment. A, Phenotypes of YZ1flowers with shade treatment were analyzed from 11 am on 0 DPA (0-11) to 6 am on 1 DPA (1-6). B, the anthocyanin contents for corresponding timepoint flowers were measured at A_530_. Error bars represent SD with three repeats performed. For each repeat, more than five flowers were analyzed.

Light has been reported to be the main environmental factor that affects anthocyanin biosynthesis. The color of cotton flowers is also significantly affected by light. However, flowers subjected to shade treatment also underwent a mild color change (Figure 3A). With shade treatment, the accumulation of anthocyanin, which should kick in at 12 am under normal light, did not increase until 6 pm on 0 DPA in YZ1 (Figure 3A). The anthocyanin content of these flowers was significantly decreased during flower development when comparing with the flower under light (Figure 2B and 3B), while it still correlated with the flower color changes (Figure 3A and B). Although the concentration of anthocyanin was reduced in the shade, a gradual accumulation pattern still showed up in these flowers, which indicated the flower color change was controlled in a light independent pattern (Figure 3B). Fertilization and pH were considered as the key factors for flower color changes, but fertilized flowers showed little difference compared to unfertilized flowers in both phenotype and anthocyanin content of YZ1 (Figure S1A and B). The pH value did not significantly change during cotton flowers development, although it was changed at 6 am on 0 DPA, and then increased at 12 am, afterwards decreased again (Figure S2). The pattern of the pH changes was not associated with the color changes during flower development (Figure 2 and S2). These results indicated that light instead of fertilization or pH was responsible for the color changes during the development of the cotton flower. Although light could affect anthocyanin accumulation, the color of the flowers also changed under shaded conditions, which indicated that the color change of cotton flower was mainly under a genetic rather than light control and light acts as an enhancer to affect the concentration of pigment.

### The expression levels of genes of the anthocyanin pathway are consistent with the color change

**Figure 4:**
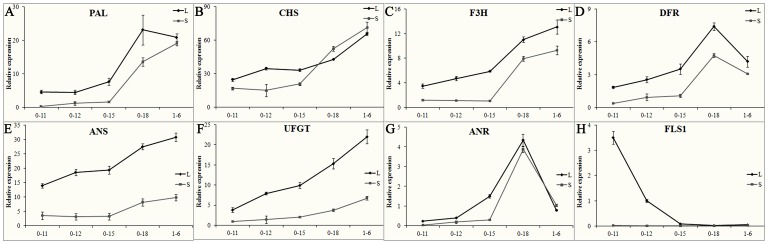
Gene expression analysis of the flavonoids biosynthesis pathway. Transcripts of *PAL* (A), *CHS* (B), *F3H* (C), *DFR* (D), *ANS* (E), *UFGT* (F), *ANR* (G) and *FLS1* (H) were analyzed by qPCR. Flowers of YZ1 with normal light (L) or in shade treatment (S) were collected from the field at five time points (from 11 am on 0 DPA (0-11) to 6 am on 1 DPA (1-6)). Transcripts were normalized with the expression of *UBQ7*. Three repeats were performed. Error bars represent SD.

Phenotypic observation of cotton flower development suggested that anthocyanin biosynthesis is controlled by both environmental and genetic factors (Figure 3). Therefore, we further studied the transcription of genes involved in this pathway (Figure 4). The transcriptions of the flavonoid and anthocyanin biosynthesis-related genes *PAL*, *CHS*, *F3H*, *DFR*, *FLS, ANR, ANS* and *UFGT* were abundant in flowers and dramatically affected by light. The expression of *CHS*, *F3H*, *ANS* and *UFGT* showed a gradual increase pattern during flower development and lower levels in shading flowers compared to that in normal light (Figure 4B, C, E and F). These findings correlated with the accumulation of anthocyanin (Figure 2 and 3 B). Furthermore, the genes *CHS* and *UFGT* also showed a corresponding expression pattern with the accumulation of anthocyanin in 3-79 and T586 flowers (Figure S3A and B). These genes may play important roles in the accumulation of anthocyanin and changes in flower color.

The expression of *FLS1*, which is related to flavonol biosynthesis, peaked at 11 am on 0 DPA (when little red color was accumulated, Figure 2A) and then decreased to a very low level at 3 pm on 0 DPA (when red color had emerged, Figure 2A) and remained the base line for the rest time points of flower development (Figure 4 H); this gene was similarly expressed in the flowers of 3-79 and T586 (Figure S3C). The expression of *FLS1* was highly light-dependent, but very few transcripts were detected in shade-treated flowers (Figure 4 H). The expression of *PAL*, *DFR* and *ANR* reached the maximum at 6 pm on 0 DPA and then sharply declined (Figure 4A, D and G). *PAL* and *DFR* showed a light-induced expression pattern, but *ANR* was not strongly affected by light (Figure 4 A, D and G). Similar expression patterns of *ANR* were also observed in 3-79 and T586 flowers, although it was not dominant in T586 flowers (Figure S3D). The expression of most genes in the flavonoid pathway was light-induced, but a similar expression pattern for these genes, except for *FLS1*, was observed during flower development regardless of light exposure. These genes expression profiles were correlated with the color changes of flower, which implies that genetic control is dominant for anthocyanin metabolism, while flavonol biosynthesis may be under a light-dependent control.

### Flavonoid is the main pigment responsible for cotton flower color

**Figure 5:**
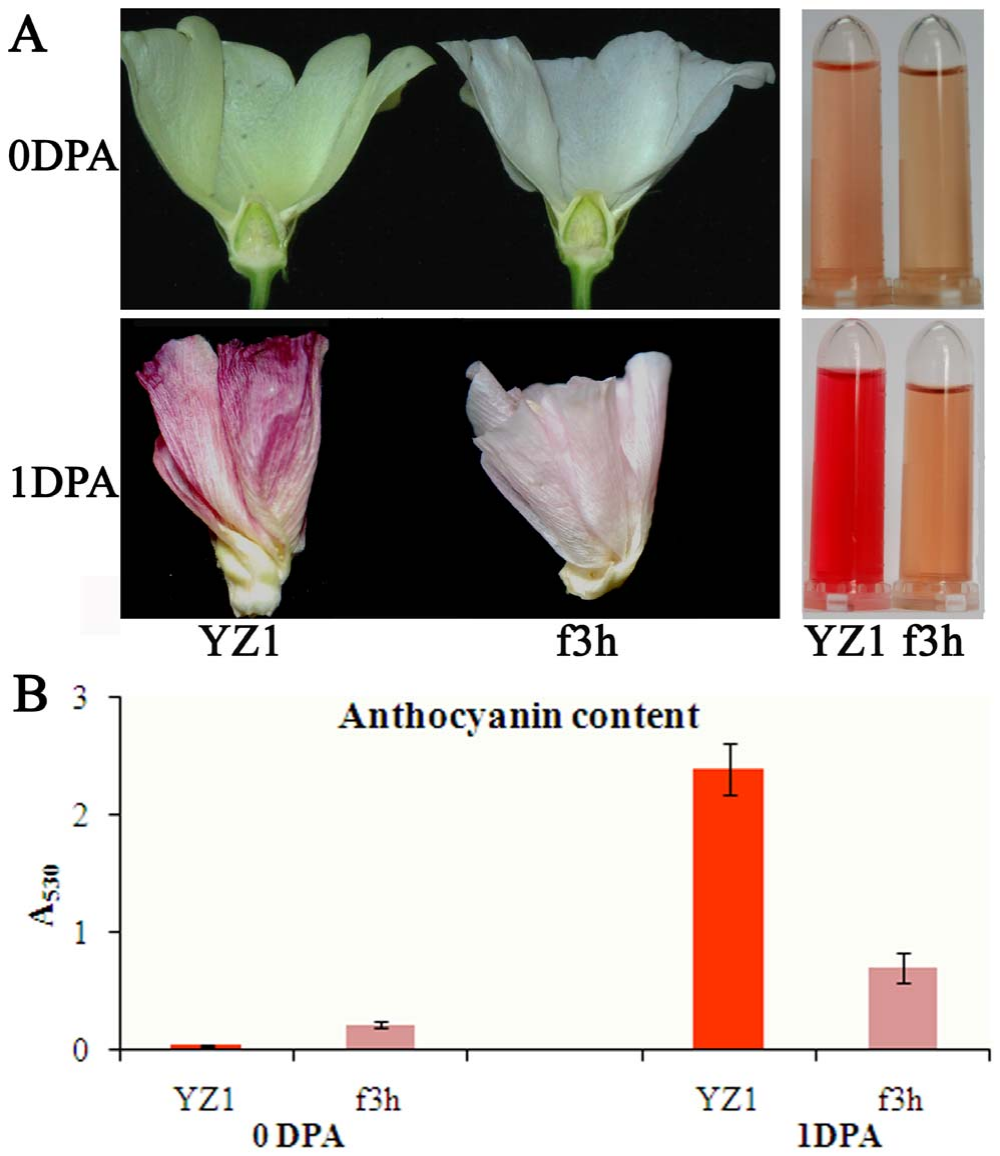
Anthocyanin analysis of YZ1 and f3h petals. A, petals of YZ1 and f3h were collected from the field at 0 and 1 DPA. The methanol extract is shown on the right side. Significant color change was observed. B, anthocyanin contents were measured at A_530_. Error bars represent SD, and three repeats were performed. For each repeat, more than five flowers were analyzed.

A previous study showed that flavonol and anthocyanin were abundant in cotton flower [36], and the silencing of the flavanone 3-hydroxylase (F3H) gene caused an obvious change in the petal color of one day post anthesis (DPA) flowers in transgenic plants [46]. To determine whether flavonoids are the main pigment in cotton flowers, we additionally compared the petals of wild type *G. hirsutum* (YZ1) and *F3H* silence line (f3h) on 0 DPA and the abscission petals on 1 DPA (Figure 5). The color of YZ1 flowers was cream at 0 DPA, which represents the most common color of cotton species, while the flower color of f3h was white (Figure 5A), which indicated that lack of flavonoids caused color loss and the pigment in the cream petals was also mainly composed of flavonoids (Figure 5). This pigment could not be anthocyanin because the OD value of the extract of YZ1 flower on 0 DPA at 530 nm was lower than that of f3h (Figure 5B). However, the fresh red extract of the abscission 1 DPA petal and the corresponding increase in OD value at 530 nm indicated that the increase in red color of the developing flower was due to anthocyanin. *F3H* silencing line had the similar color changes as previously described for non-abscission 1 DPA petals [46]. These results confirmed that flavonoids are the main pigment responsible for cotton flower color including red, cream and white.

### Genetic and environmental interactions control flavonoid metabolism in cotton flower

**Figure 6:**
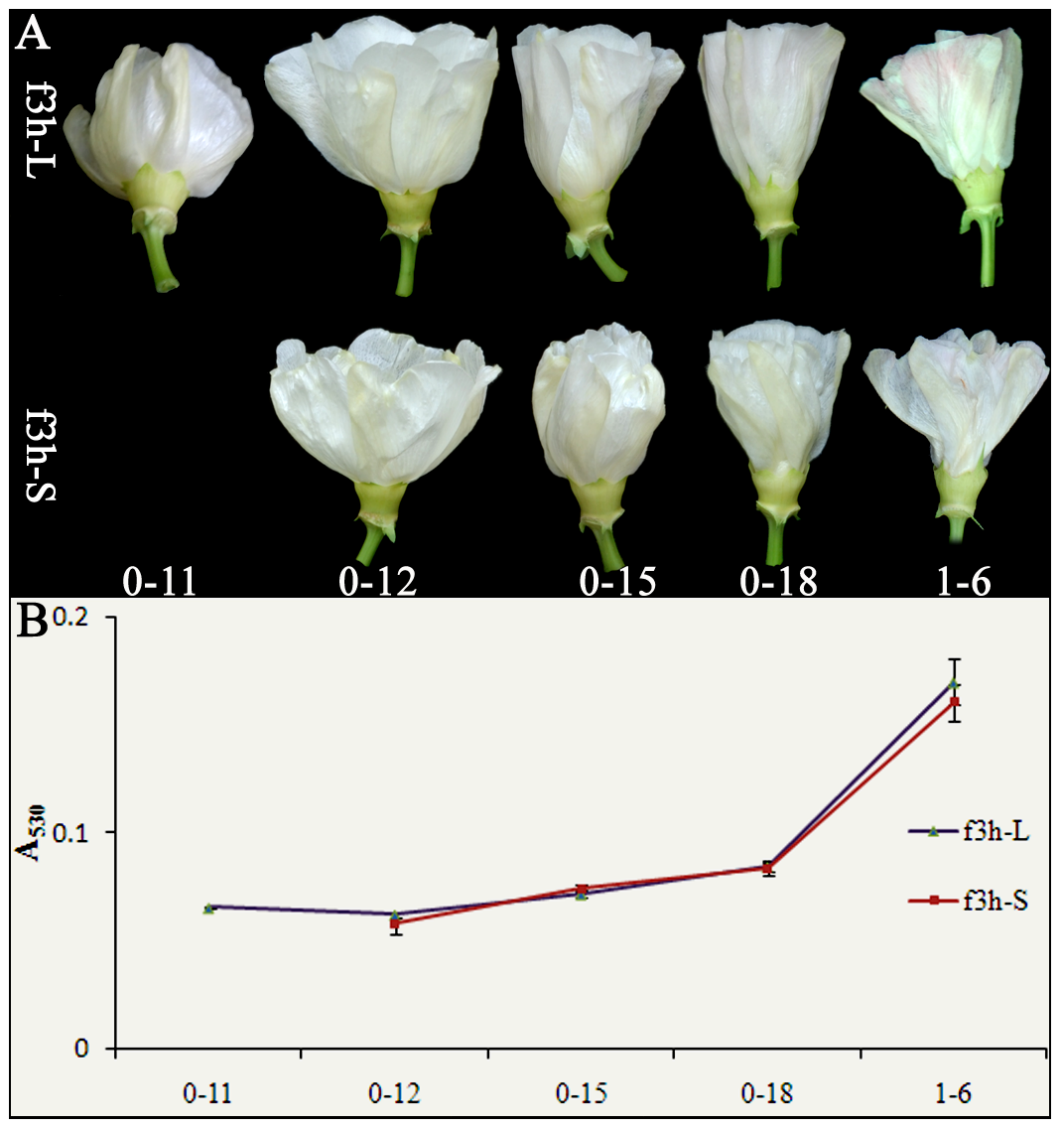
Flower color is affected by light and gene interaction. A, flowers of f3h lines were analyzed from 11 am on 0 DPA (0-11) to 6 am on 1 DPA (1-6). Flowers that were treated with normal light or shade are indicated by ‘-L’ and ‘-S’, respectively. B, anthocyanin contents for corresponding flowers were measured at A_530_. Error bars represent SD, and three repeats were performed. For each repeat, more than five flowers were analyzed.

**Figure 7:**
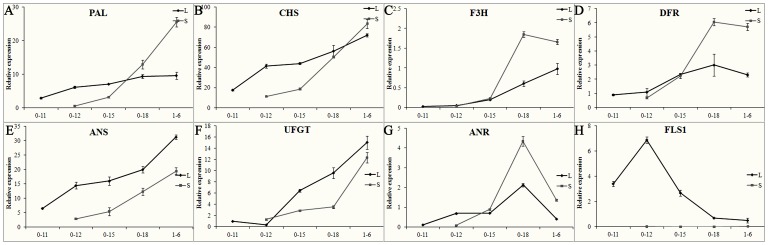
Gene expression of the flavonoids biosynthesis pathway was affected by both shade and *F3H* silence. Transcripts of *PAL* (A), *CHS* (B), *F3H* (C), *DFR* (D), *ANS* (E), *UFGT* (F), *ANR* (G) and *FLS1* (H) were analyzed by qPCR. Flowers of f3h with normal light (L) or in shade treatment (S) were collected from the field at five time points (from 11 am on 0 DPA (0-11) to 6 am on 1 DPA (1-6)). Transcripts were normalized with the expression of *UBQ7*, and three repeats were performed. Error bars represent SD.

The flower color of the f3h line changed during its development. A red color appeared on 1 DPA in the f3h line which was significantly delayed compared to YZ1, which emerged at 12 am on 0 DPA (Figure 6A). The f3h line showed a slight decrease at 6 am on 1 DPA in anthocyanin accumulation following shade treatment compared to non-treatment (Figure 6B). Gene expression analysis showed that the expression pattern of these flavonoid genes was similar in the f3h and YZ1 lines during flower development, however, the expression of *PAL*, *DFR, UFGT* and *ANR* was suppressed by the silence of *F3H* (Figure 4 and 7). Shade also significantly affected gene expression in the f3h line, while *PAL*, *F3H, DFR* and *ANR* were all expressed at higher levels after 3 pm on 0 DPA when grown under the shade treatment compared to normal light, at that time the anthocyanin was dramatically accumulated in the YZ1 flower (Figure 7A, C, D and G). All of these flavonoid genes showed higher expression levels at 6 am on 1 DPA in the f3h line compared to YZ1 with shade treatment (Figure 4 and 7), at that time point anthocyanin was abundantly accumulated in the YZ1 flower (Figure 2). These results showed that there was a complicated interaction between gene expression and light exposure, especially for *PAL*, *DFR* and *ANR*, which were indirectly related with anthocyanin biosynthesis. These genes were all reduced by either shade treatment or silence of *F3H*, but they showed similar expression levels in the shade treated f3h line as YZ1 under normal light treatment.

Additionally, the expression of *FLS1* was strictly light dependent in f3h flowers (Figure 7H). Silence of *F3H* caused a maximum expression level at 12 am on 0 DPA, although it showed a similar expression level at 11 am as that of YZ1 under normal light (Figure 4H). Afterwards, the expression of *FLS1* decreased but still maintained relatively higher expression levels compared to YZ1 (Figure 7H). We further investigated two additional FLS genes, identified as *FLS2* and *FLS3*, from the published cotton D genome. *FLS3* showed a more dominant expression in flowers, similar to *FLS1*, compared with *FLS2* (Figure S4A and B). *FLS2* and *FLS3* also showed a peak expression at 11 am and then decreased in YZ1, 3-79 and T586 (Figure S4A and B). Light and silence of *F3H* showed less effect on the expression of *FLS2*, although the expression of *FLS2* was also enhanced in the flower of f3h silencing lines (Figure S4A). On the other side, the expression of *FLS3* showed a similar expression pattern as that of *FLS1*, which was significantly affected by light and showed a higher and delayed maximum expression at 12 am on 0 DPA in flowers f3h lines compared to YZ1 (Figure S4B). The significant effect of light on the expression of the dominantly expressed *FLS1* and *FLS3* indicates that the flavonol pathway was mainly light dependent in cotton flowers, and silence of *F3H* could increase the expression of *FLS* under light exposure. These results imply that flavonol is not associated with flower color changes but has a significant role under light response.

## Discussion

The cotton flowers have been studied for more than a century. Although many different flower colors have been observed, it has been shown that the pigments of cotton flower mostly contain flavonols [35]. Yellow flowers of *Gossypium herbaceum* and *G. barbadense* contain gossypitrin, isoquercitrin quercimeritrin and herbacitrin, and the red flowers of *G. arboreum* also contain isoquercitrin [35], [50]. Throughout many years of introduction and breeding, the major cultivated and collected cotton germplasms were consisted of *G. hirsutum*, and the major color of cotton flower was cream (Figure 1). However, the pigment responsible for the cream color is also composed of flavonoids, and likely also flavonols, because silence of *F3H* in cotton results in white flowers (Figure 5A). The extract of red cotton flowers showed a high OD value at 530 nm, which indicates high anthocyanin content (Figure 2 and 5). All of these results confirmed that the color of cotton flowers is mainly related to flavonoids content.

The color of flavonoids is not stable and changes as the result of differences in pH and ion concentration [3], [4]. The cotton flowers undergo dramatic color changes during their short lifespan (Figure 2). It has been hypothesized that these color changes could correlate with changes in the environment. Our results indicated that the color change was not primarily associated with pH variation but showed a strong relationship with the expression of anthocyanin biosynthesis genes (Figure 4 and S2). The results also showed that the accumulation of anthocyanin is responsible for the color changes (Figure 2 and 3). The color change is an endogenous genetic process rather than solely the effect of environment, which was in correspondence with the previous findings showing that the constituents of cotton flower petal appear to be less variable as a result of environmental effects [39]. Light also play an important role in color changes of cotton flower, and the flower under shade treatment showed decreased and delayed anthocyanin accumulation (Figure 3). However, light only had limited effects on anthocyanin accumulation, as the blooming flowers under shade treatment showed the same cream color as those under normal light (Figure 3A), while the f3h lines showed white petals in both normal light and shading treatment during flower development (Figure 6A). Additionally, all flowers including F3H silencing lines with or without light exposure showed the same patterns of anthocyanin accumulation (Figure 3B and 6B). Gene expression analysis also showed the same expression pattern between flowers under light and shading treatment (Figure 4). These results further confirmed that the color change of cotton flowers is genetically controlled (Figure 3). The transcription of *ANR* genes that respond to proanthocyanidin biosynthesis was showed to be specially time-point regulated, with a gradual increase from 11 am to a peak expression at 6 pm on 0 DPA; however, *ANR* showed less light response than the other genes (Figure 4G and S3D) and may not respond to the flower color change. Silence of *F3H* could significantly affect the expression of flavonoid genes, especially *PAL*, *DFR*, and *ANR*, which showed co-suppression with *F3H* (Figure 4 and 7). All of these genes showed higher expression levels in f3h lines following shade treatment compared to normal light exposure after 3 pm (Figure 7), when anthocyanin was beginning to accumulate in normal flower. The results indicated that there was a feedback control for these genes under normal conditions. The excess accumulation of flavonoids upstream of F3H or the deficiency of chemicals downstream of F3H could suppress the expression of *PAL*, *DFR* and *ANR* under light exposure. However, in shade treatment, the levels of excess flavonoids and deficient chemicals in the f3h lines should be less different with that of YZ1 comparing with that under light exposure. These results imply that there is a complex interaction between light and endogenous genetic factors to affect the flower color biosynthesis. It was different from that in *Viola cornuta* L. (Violaceae) flowers whose color change was totally light dependent [12].

Flavonol and anthocyanin were the main flavonoids found in the cotton flower. The accumulation of anthocyanin and the expression of anthocyanin biosynthesisrelated genes were gradually increased from 12 am on 0 DPA (Figure 2, 3 and 4). For flavonols, the expression of *FLS1* also showed light exposure controlled (Figure 4H and 7H). The highest transcriptional level of *FLS1* occurred at 11 am in all the three cotton species and was then dramatically decreased to a very low level by 3 pm (Figure S3). Silence of *F3H* not only increased the expression of *FLS* genes but also delayed the highest expression level at 12 am (Figure 7H and S4). These findings indicate that there is a light driven flavonols biosynthesis procedure during flower blooming in the morning of 0 DPA. Once this procedure was activated and suitable FLS proteins and flavonols were produced, the expression of *FLS* genes would be decreased. If the level of flavonols was insufficient under light exposure, such as what occurred in the *F3H* silenced flowers, then there was a feedback procedure to increase the expression of *FLS* genes to produce higher levels of flavonols. Additionally, the feedback procedure was strongest at 12 am and medially lasted to 6 pm of 0 DPA (Figure 7H). The two dominantly expressed *FLS* genes, *FLS1* and *FLS3*, were showed to be at a higher expression level at 12 am in the f3h lines and maintained a moderate level of expression afterwards, when low transcripts level of *FLS* genes were detected in YZ1 flowers (Figure 4H, 7H and S4B). According to the expression levels of flavonoid genes, a special flavonoid flux mechanism was present during cotton flower development. The previous high expression levels of *FLS* genes at 11 am made the flavonoid metabolism switch to flavonol biosynthesis in response to light exposure. However, it is still not clear about the mechanisms of the metabolic flux into proanthocyandin biosynthesis at 6 pm on 0 DPA and the final metabolic flux into anthocyanin biosynthesis at the end of the flower life in 1DPA.

Cotton is not a model plant and its genome is complex, so its genetic manipulation is difficult, even though one cotton genome has been sequenced [51], [52]. However, the study of the short developmental life span and special genetic regulation of flavonoid biosynthesis in cotton flower would benefit to understand the flower development. Although fiber is the main product of cotton, the cotton flower could be engineered to improve the economic value of cotton. The yield of cotton flowers is far greater than that of cotton fiber; therefore, there is a great potential for cotton flowers to yield by-products, including abundant flavonoids and sugars, which would be beneficial for the chemical industry and agriculture. Furthermore, the cotton flower is the primary target of the cotton bollworm, which accounts for substantial yield losses in the cotton field [53]. Traditional genetically modified cotton cannot guarantee a persistent level of toxic BT proteins in the cotton flower, which would increase the survival of bollworm larvae [54]. Thus the cotton flower could be manipulated to protect against invasion by cotton bollworms. Our findings on the factors and regulations of the flavonoid pathways during the development of cotton flower color would provide a starting point for such studies.

## Supporting Information

Figure S1 **Effect of fertilization on the accumulation of anthocyanin in cotton flowers.** A, normally fertilized (Y-F) and emasculated YZ1 flowers (Y-U) were collected from the field at 8 am on 1 DPA. B, anthocyanins of the Y-F and Y-U flowers were measured at A_530_. Three repeats were performed. Error bars represent SD.
(TIF)

Figure S2 **pH values of the developing cotton flowers.** YZ1 and f3h flowers taken from five time points between 6 pm on -1 DPA (-1-18) and 6 am on 1 DPA (1-6) were collected and analyzed. Three repeats were performed. Error bars represent SD.
(TIF)

Figure S3 **Expression pattern of flavonoid genes in developmental flowers of 3-79 and T586.** The transcripts of *CHS* (A), *UFGT* (B), *FLS1* (C) and *ANR* (D) were analyzed at five time points (from 11 am, 12 am, 3 pm and 6 pm of 0 DPA (0-11, 0-12, 0-15 and 0-18) to 6 am on 1 DPA (1-6)). Transcripts were normalized with the expression of *UBQ7*. Three repeats were performed. Error bars represent SD.
(TIF)

Figure S4 **Expression analysis of FLS2 and FLS3 in cotton flowers.** Expression analysis of *FLS2* (A) and *FLS3* (B) in flowers of YZ1-L, shade-treated YZ1 (YZ1-S), f3h, 3-79 and T586 was performed with qPCR. Flowers at 11 am (0-11), 12 am (0-12) and 3 pm (0-15) on 0 DPA were collected for analysis. Transcripts were normalized with the expression of *UBQ7*. Three repeats were performed. Error bars represent SD.
(TIF)

Table S1 **Primers used in this study.**
(DOC)
