# Supplementary figures and images for: The Flavonoid Pathway Regulates the Petal Colors of Cotton Flower
Source: PLoS One. 2013 Aug 12;8(8):e72364. doi: 10.1371/journal.pone.0072364 (PMC3741151; doi:10.1371/journal.pone.0072364)

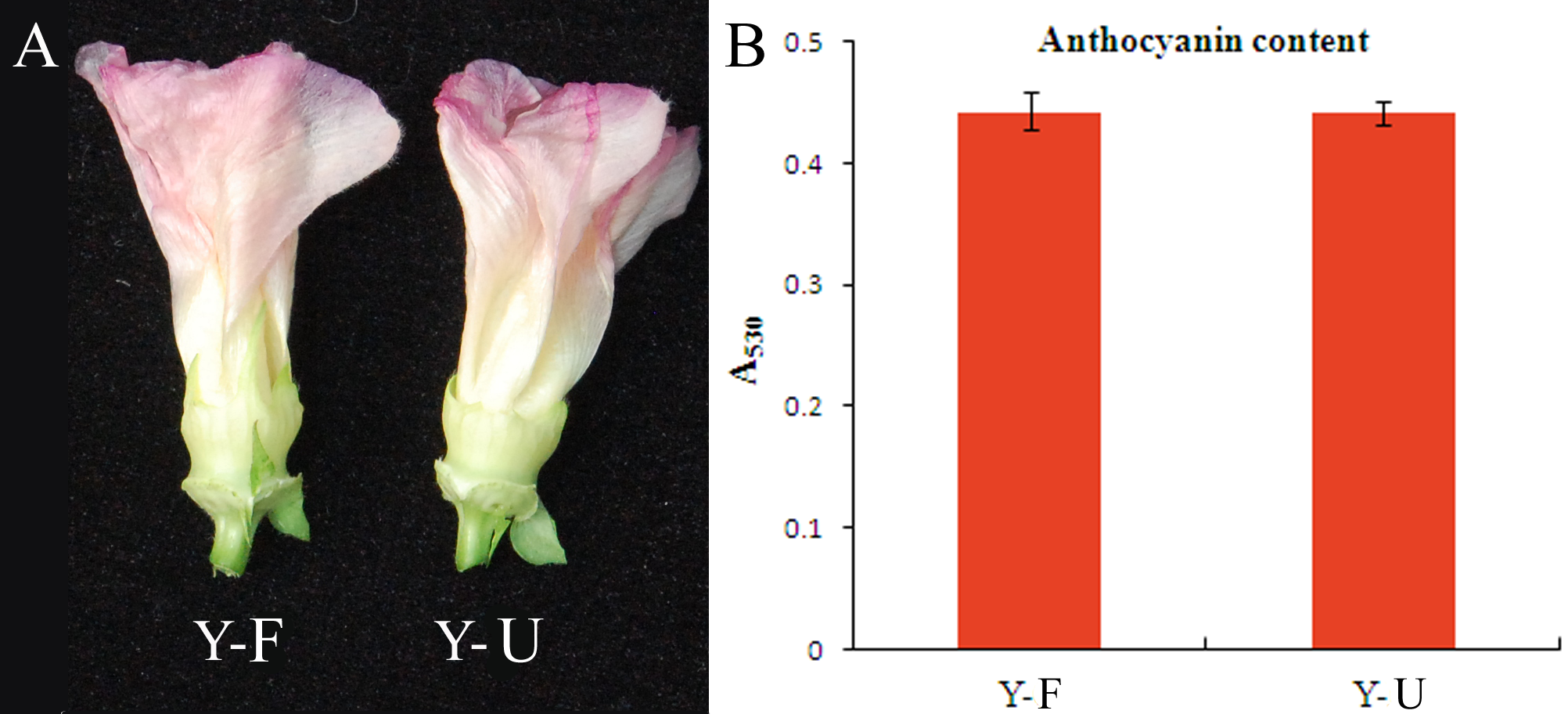

Supplement: Figure S1 — Effect of fertilization on the accumulation of anthocyanin in cotton flowers. A, normally fertilized (Y-F) and emasculated YZ1 flowers (Y-U) were collected from the field at 8 am on 1 DPA. B, anthocyanins of the Y-F and Y-U flowers were measured at A530. Three repeats were performed. Error bars represent SD. (TIF) [file pone.0072364.s001.tif]

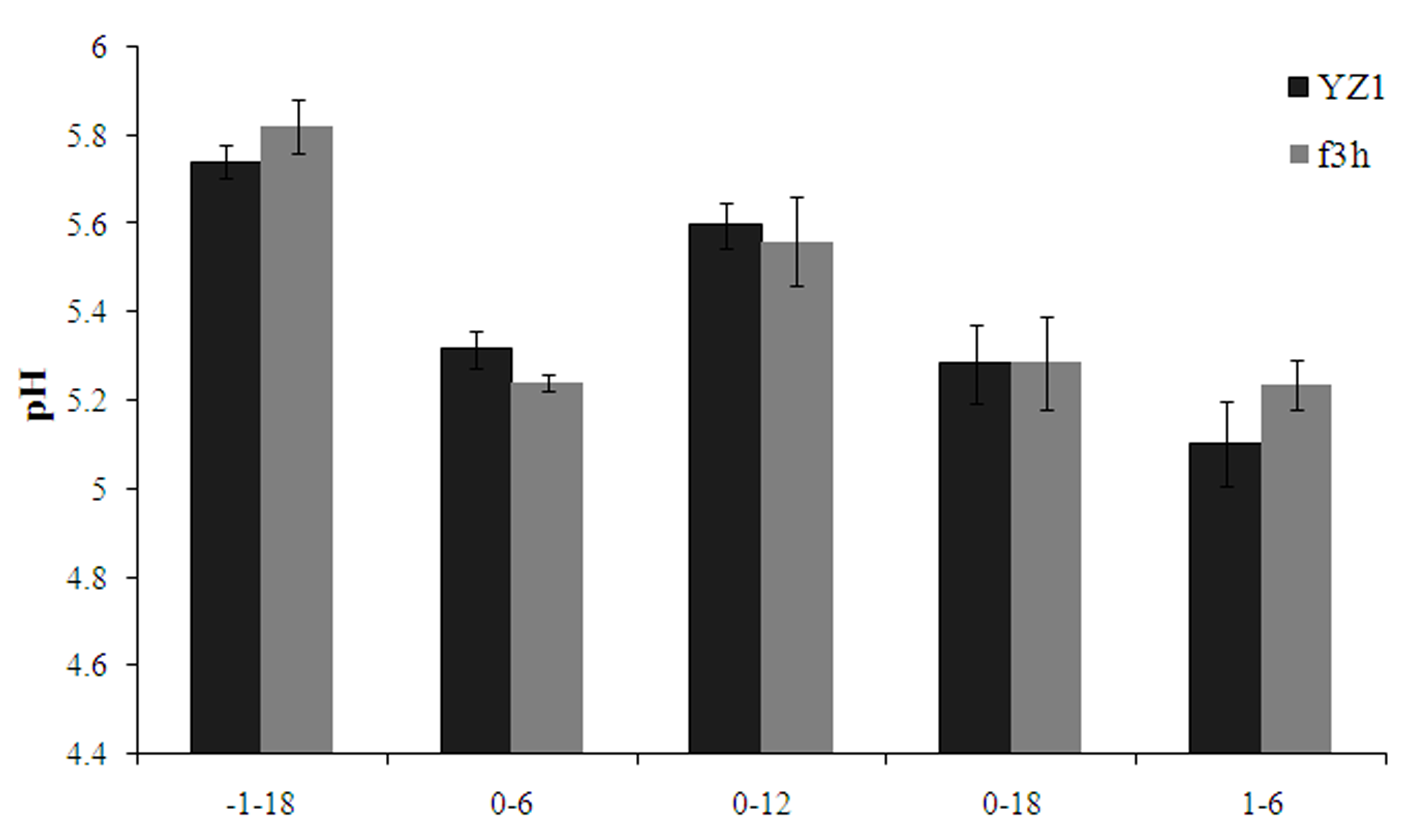

Supplement: Figure S2 — pH values of the developing cotton flowers. YZ1 and f3h flowers taken from five time points between 6 pm on -1 DPA (-1-18) and 6 am on 1 DPA (1-6) were collected and analyzed. Three repeats were performed. Error bars represent SD. (TIF) [file pone.0072364.s002.tif]

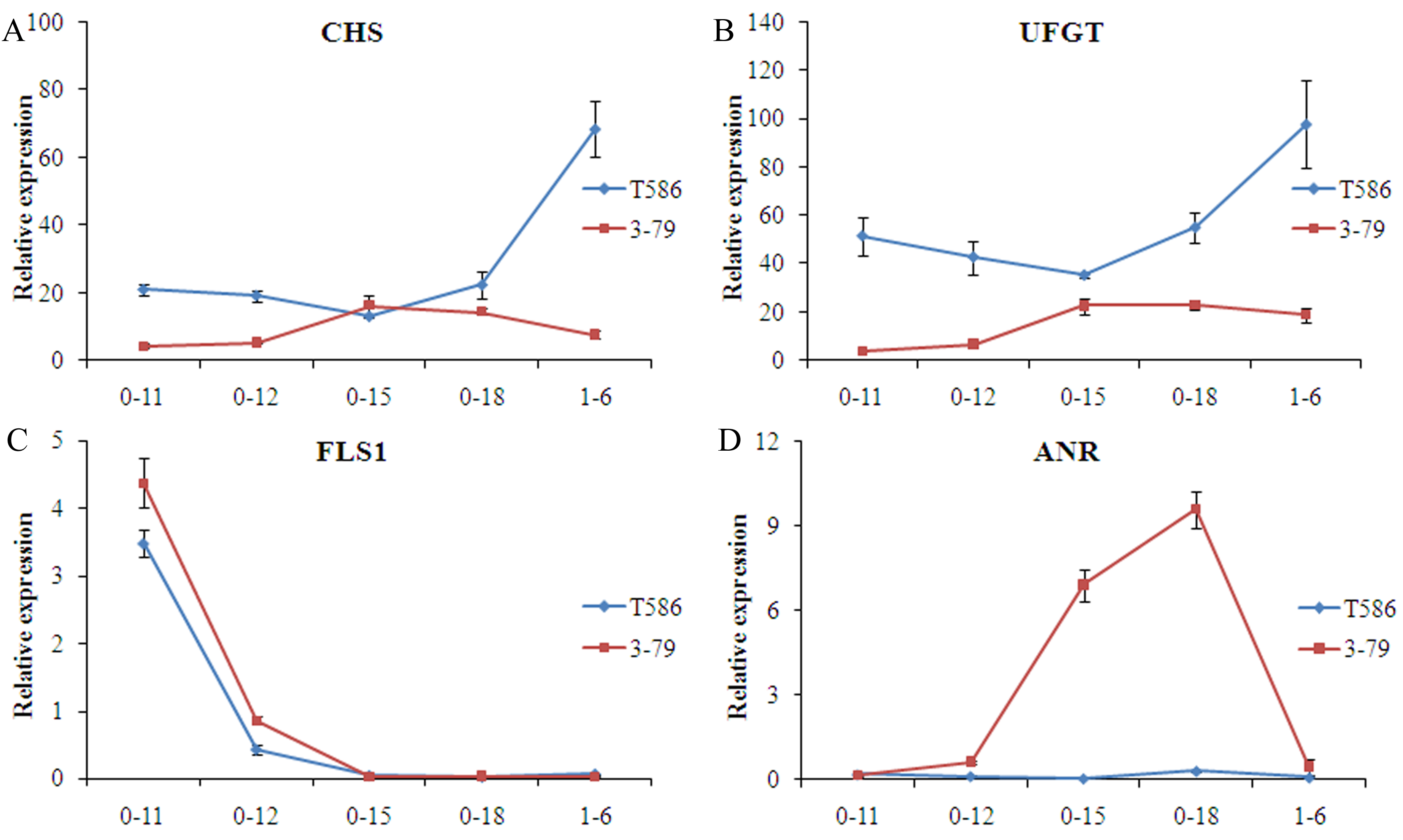

Supplement: Figure S3 — Expression pattern of flavonoid genes in developmental flowers of 3-79 and T586. The transcripts of CHS (A), UFGT (B), FLS1 (C) and ANR (D) were analyzed at five time points (from 11 am, 12 am, 3 pm and 6 pm of 0 DPA (0-11, 0-12, 0-15 and 0-18) to 6 am on 1 DPA (1-6)). Transcripts were normalized with the expression of UBQ7. Three repeats were performed. Error bars represent SD. (TIF) [file pone.0072364.s003.tif]

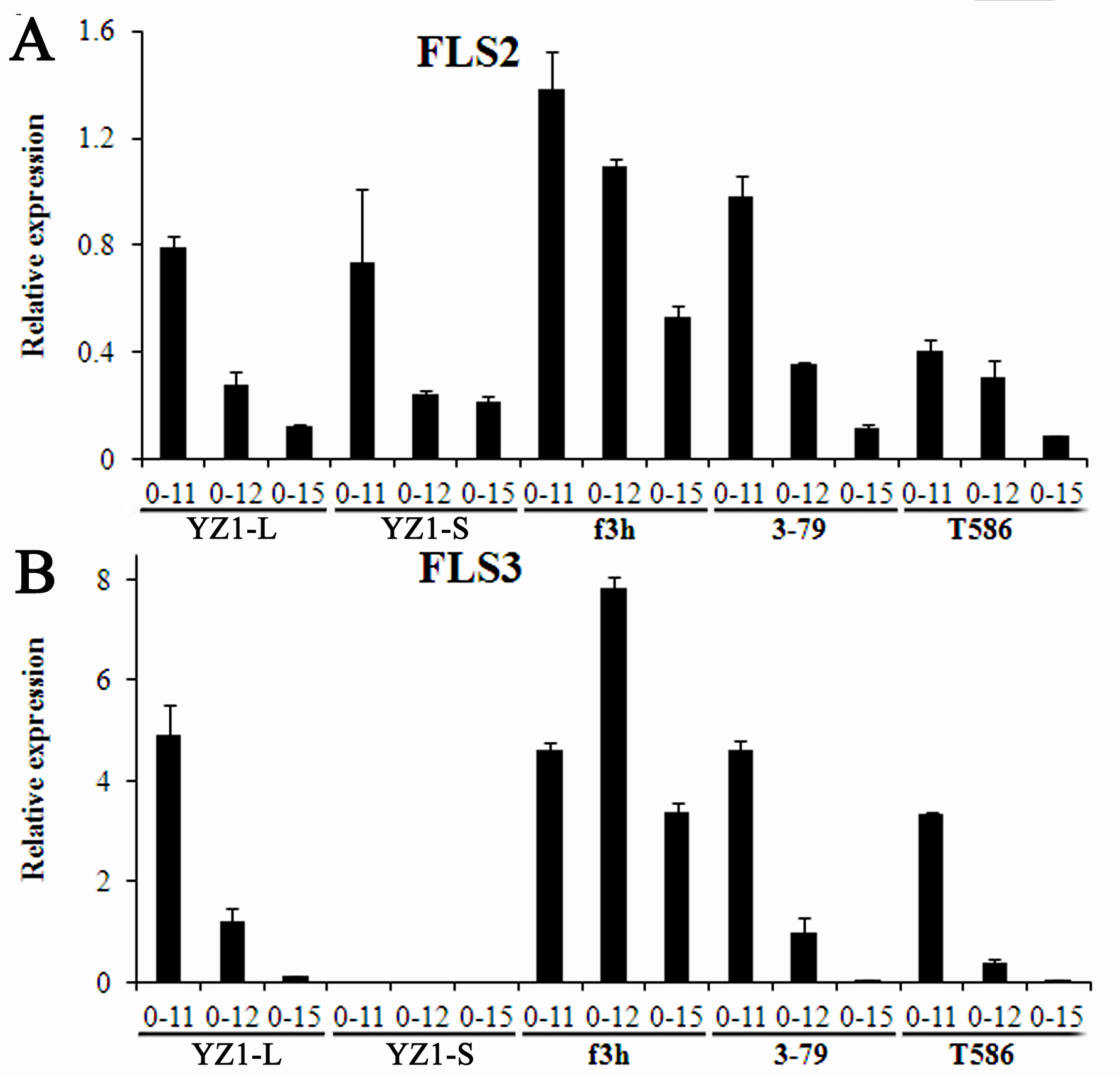

Supplement: Figure S4 — Expression analysis of FLS2 and FLS3 in cotton flowers. Expression analysis of FLS2 (A) and FLS3 (B) in flowers of YZ1-L, shade-treated YZ1 (YZ1-S), f3h, 3-79 and T586 was performed with qPCR. Flowers at 11 am (0-11), 12 am (0-12) and 3 pm (0-15) on 0 DPA were collected for analysis. Transcripts were normalized with the expression of UBQ7. Three repeats were performed. Error bars represent SD. (TIF) [file pone.0072364.s004.tif]
