# Supplementary material for: The Flavonoid Pathway Regulates the Petal Colors of Cotton Flower
Source: PLoS One. 2013 Aug 12;8(8):e72364. doi: 10.1371/journal.pone.0072364 (PMC3741151; doi:10.1371/journal.pone.0072364)
Supplement: Table S1 — Primers used in this study. (DOC) [file pone.0072364.s005.doc]

Table S1 primers used in this study

| Gene | Gene Description | Gene ID ^a^ | Sense Primer 1 (5′–3′)  Anti-sense Primer 2 (5′–3′) | |
| --- | --- | --- | --- | --- |
| PAL | phenylalanine ammonia-lyase | JN032297 | | 1: CCCATCAAACCTCTCCGCTAGT  2: TGCTTCGGCTGTTTTTCGTG |
| CHS | chalcone synthase | Ghi.1443 | | 1:TGTGTGCTCGGAGATTACTGCT  2:GTTCAAACATGGGCTTCTCGAT |
| F3H | flavanone 3-hydroxylase | Ghi.17176 | | 1:GGGCCTAGCTTGCAAGCTTCTT  2:AAGCAAGAGTGTGATGGTGCCTGG |
| DFR | dihydroflavonol 4-reductase | Ghi.9795 | | 1:CGAGGACCCTGAGAATGAAGT  2:GGCTTTTGTTGTTCTGCAACAT |
| ANS | anthocyanidin synthase | Ghi.1234 | | 1:GAGGCCTAGCGAGCAAAATAC  2:GTGAGCTTCGACACCGAGAG |
| UFGT | UDP-glucose:flavonoid 3-O-glucosyltransferase | Ghi.12023 | | 1: TGTTCTCGCATTCCCTCACG  2: CCTCCGGGTACTCCATCGTC |
| ANR | anthocyanidin reductase | Ghi.8422 | | 1:CCGCAGCTGTGTCTATCAACAC  2:AGCAAATTTCCAAGCTGCCTT |
| FLS1 | flavonol synthase 1 | Gorai.012G026100.1 | | 1: CAAGTTGTGAACCATGGCATTC  2: GCCTTCAATGGATTGAGATCCT |
| FLS2 | flavonol synthase 2 | Gorai.009G243600.1 | | 1: TACTATCCGCAGTGCCCTCG  2: TTCCGTTGCTCACAATCTCG |
| FLS3 | flavonol synthase 3 | Gorai.012G026200.1 | | 1: GTGTTGGTGCCCAACGAGGT  2: CCCCTGGAGGCTCCAAGAAT |
| UBQ7^b^ | ubiquitin 7 | DQ116441 | | 1:GAAGGCATTCCACCTGACCAAC  2:CTTGACCTTCTTCTTCTTGTGCTTG |

^a^This primer was designed according to the conserved region of the sequences from the NCBI UniGene data bank and the D-genome sequences.

^b^Primers are from previous papers ([1](#_ENREF_46)).

1. Tu LL, Zhang XL, Liang SG, Liu DQ, Zhu LF, et al. (2007) Genes expression analyses of sea-island cotton (*Gossypium barbadense* L.) during fiber development. Plant Cell Rep 26: 1309-1320.
